# Effects of gait retraining with focus on impact versus gait retraining with focus on cadence on pain, function and lower limb kinematics in runners with patellofemoral pain: Protocol of a randomized, blinded, parallel group trial with 6-month follow-up

**DOI:** 10.1371/journal.pone.0250965

**Published:** 2021-05-12

**Authors:** José Roberto de Souza Júnior, Pedro Henrique Reis Rabelo, Thiago Vilela Lemos, Jean-Francois Esculier, João Pedro da Silva Carto, João Paulo Chieregato Matheus

**Affiliations:** 1 Sciences and Technologies in Health Post-graduation Program, University of Brasília, Brasília, Federal District, Brazil; 2 Department of Physical Therapy, State University of Goiás, Goiânia, Goiás, Brazil; 3 The Running Clinic, Lac Beauport, Quebec, Canada; 4 Department of Physical Therapy, University of British Columbia, Vancouver, British Columbia, Canada; Prince Sattam Bin Abdulaziz University, College of Applied Medical Sciences, SAUDI ARABIA

## Abstract

Patellofemoral pain (PFP) is one of the most prevalent injuries in runners. Unfortunately, a substantial part of injured athletes do not recover fully from PFP in the long-term. Although previous studies have shown positive effects of gait retraining in this condition, retraining protocols often lack clinical applicability because they are time-consuming, costly for patients and require a treadmill. The primary objective of this study will be to compare the effects of two different two-week partially supervised gait retraining programs, with a control intervention; on pain, function and lower limb kinematics of runners with PFP. It will be a single-blind randomized clinical trial with six-month follow-up. The study will be composed of three groups: a group focusing on impact (group A), a group focusing on cadence (group B), and a control group that will not perform any intervention (group C). The primary outcome measure will be pain assessed using the Visual Analog Pain scale during running. Secondary outcomes will include pain during daily activities (usual), symptoms assessed using the Patellofemoral Disorders Scale and lower limb running kinematics in the frontal (contralateral pelvic drop; hip adduction) and sagittal planes (foot inclination; tibia inclination; ankle dorsiflexion; knee flexion) assessed using the MyoResearch 3.14—MyoVideo (Noraxon U.S.A. Inc.). The study outcomes will be evaluated before (t0), immediately after (t2), and six months (t24) after starting the protocol. Our hypothesis is that both partially supervised gait retraining programs will be more effective in reducing pain, improving symptoms, and modifying lower limb kinematics during running compared with the control group, and that the positive effects from these programs will persist for six months. Also, we believe that one gait retraining group will not be superior to the other. Results from this study will help improve care in runners with PFP, while maximizing clinical applicability as well as time and cost-effectiveness.

## Introduction

Millions of people practice running, an activity that requires minimal equipment and benefits cardiac, metabolic, and mental health. However, running tends to cause high injury rates (19% to 92%) leading to social and economic losses [1–5]. In runners, the knee is the main site affected (7.2% to 50%) [5] and one of the main injuries is patellofemoral pain (PFP) (5.5%) [6]. Unfortunately, 55% of treated individuals present unfavourable recovery at three months and 40% at twelve months [7]. Also, 57% do not recover completely in five to eight years [8].

Various interventions are recommended for the treatment of individuals with PFP, among which gait retraining can be highlighted [9]. A systematic review by Agresta and Brown [10], demonstrated immediate and short-term effects (1 and 3 months) on pain, function, and biomechanics of individuals with PFP, results corroborated by Barton and colleagues [11], who associated current evidence with expert opinions. Findings were also outlined by critical reviews conducted by Jeon and Thomas [12] and Davis and colleagues [13].

However, the evidence of the benefits of gait retraining comes mainly from case series and quasi-experimental studies that did not have a control group and did not report adverse effects or limiting factors for this intervention [10–12]. The need for high-quality evidence justifies conducting clinical trials that show the effects of gait retraining programs in individuals with PFP.

The systematic review and meta-analysis of Neal and colleagues [14], found moderate evidence that runners with PFP had increased hip adduction and contralateral pelvic drop, based on cross-sectional studies. Reducing peak knee flexion could also be an important variable to address in runners with PFP, given that greater flexion has been associated with greater patellofemoral joint loading [15]. Increasing running cadence has been shown to be effective in reducing hip adduction [16,17], contralateral pelvic drop [16], and knee flexion [16,17] in subjects with PFP. Three additional studies show that increasing cadence reduces compression forces in the patellofemoral joint [15,18,19]. Another strategy with interesting results is modifying knee joint loading through visual and verbal feedback on impact. Indeed, two studies found reductions in ground reaction force loading rates using specific cues on “run softer” and an accelerometer [20,21], and this has been shown to reduce the incidence of PFP [22].

Unfortunately, the main gait retraining protocols are not feasible in clinical practice, since they consume a lot of time and financial resources for patients [11,23]. In fact, a recent study showed positive effects after a single session of cadence retraining on symptoms and biomechanics of runners with PFP [16]. Therefore, we chose to compare two partially supervised running retraining programs, one group focusing on impact and another group focusing on cadence.

The primary objective of the study will be to compare the effects of two different two-week partially supervised gait retraining programs, one focusing on impact and the other focusing on cadence, with a control intervention; on pain, function and lower limb kinematics of runners with PFP. The secondary objective of the study will be to compare the effects of these programs on pain, function and lower limb kinematics six months after their application.

Gait retraining with focus on impact has been proved to modify the ground reaction forces in pain free-subjects (tibial acceleration, vertical impact peak, instantaneous and average loading rates) [20–22], while the gait retraining with focus on cadence has been proved to modify symptoms, kinematic (increased hip adduction, contralateral pelvic drop and knee flexion) and kinetic aspects (patellofemoral loading rate) in patellofemoral pain patients [15,16]. Our hypothesis is that the two partially supervised gait retraining programs will be more effective in reducing pain, improving symptoms, and modifying lower limb kinematics during running compared with the control group, and that the positive effects from these programs will persist for six months. It is expected a reduction in contralateral pelvic drop, hip adduction, knee flexion, ankle dorsiflexion, tibia and foot inclination in both retraining groups. Also, we believe that one gait retraining group will not be superior to the other. The main motivations to perform a comparison of partially supervised gait retraining programs are the lack of clinical trials and the possibility to provide viable options for runners with PFP.

## Methods

### Study design

This will be a randomized, single-blind, parallel group, three-arm superiority clinical trial, using a 1:1:1 allocation rate and six-month follow-up. The three arms of the study will be composed of three groups: a group that will perform partially supervised gait retraining with a focus on impact (group A), a group that will perform partially supervised gait retraining focusing on cadence (group B), and a control group that will not perform any intervention (group C). The study outcomes will be evaluated before (t0), immediately after (t2), and six months (t24) after starting the protocol. The study will be carried out at the Instituto Trata located in the city of Goiânia, Goiás, Brazil. This protocol was written in accordance with the SPIRIT (Standard Protocol Items: Recommendations for Intervention Trials) (S1 and S5 Files) and the results will be reported in accordance with the CONSORT (Consolidated Standards Of Reporting Trials). Information regarding the dissemination of the results can be found in S2 File.

### Participants

The eligibility criteria will be: Inclusion–(I) rearfoot striking runners with cadence under 170 steps/minute; (II) age between 18 and 45 years; (III) pain around or behind the patella, with intensity of at least 3/10 on the Visual Analogue Scale (VAS), during running and one task among squatting, climbing, and descending steps, kneeling and extending the knee with resistance [24]; (IV) being comfortable running at a speed of 10–12 km/hour. Exclusion—(I) Other disorders in the lower limbs; (II) history of surgery in the lower limbs in the last year; (III) not showing interest to adhere to a strict running retraining protocol for 2 weeks. Participants with unilateral or bilateral symptoms will be included, however, in the second case, only the worst knee (pain intensity) will be considered for analysis. The participants will be recruited through contact via e-mail, telephone, and through personnel with Goiânia’s running clubs in search of runners with PFP. In this first contact, general (gender, age, body mass, height, body mass index, history of injury and previous surgeries in the lower limbs), running (experience, volume, frequency) and pain information (intensity, location, duration of symptoms and functional tasks related to pain) will be assessed to see if the participants are eligible. Based on pain information, the diagnostic will be established using the following criteria: pain around or behind the patella during functional activities that loads the patellofemoral joint [24]. A systematic review of reviews concluded that anterior knee pain produced by functional tasks is currently the best diagnostic indicator of PFP [25]. Also, PFP is evident in 80% of the people who present anterior knee pain during a squatting manoeuvre [26].

Ethics approval was obtained from the Institutional Review Board of University of Brasilia/Faculty of Ceilândia (4.132.491), and all subjects will sign a detailed consent form before entering the study (S2–S4 Files). This study was registered in the Brazilian Registry of Clinical Trials (RBR-8yb47v) (S6 File).

### Sample size

An a priori sample size calculation was conducted using the eta-partial square value of the group-by-time interaction for worst knee pain (0.19) obtained in a previous study that compared the effects of three gait retraining programs on runners with patellofemoral pain [27]. In G*Power software [28], we used ANOVA: repeated measures, within-between interaction, with the following parameters: effect size f = 0.48 (obtained through eta-partial square value); level of confidence = 0.05; power = 99%; number of groups = 3; number of measurements = 3. The total sample size obtained was 21 participants, however, to account for losses during the follow-up period, the final sample size will be 30 participants.

### Randomisation/Blinding

Participants will be randomized into experimental or control groups with an allocation ratio of 1:1:1 by means of block randomization (block size of 2x15) performed with the aid of a sequence of numbers generated on a computer. The allocation will be hidden by means of opaque envelopes, sealed and numbered consecutively. A laboratory employee who will not participate in the evaluations and interventions will generate the allocation sequence, hide the allocation and allocate participants for interventions. Due to the nature of the intervention, both the participants and the researcher responsible for the gait retraining cannot be blind to the allocation of interventions; however they will be strongly warned not to confide the allocation in subsequent evaluations and instructed to perform the retraining sessions alone. In this way, the risk of the blinded-assessor and other participants knowing what retraining is being performed is diminished. Also, the main researcher who will conduct data analysis will be blinded to assigned group.

### Data collection

Pain will be assessed using the Visual Analog Pain Scale [29], which consists of a numerical scale from 0 to 10 points, where 0 means no pain and 10 means the maximum pain ever experienced. Pain during daily activities (usual) and running will be measured. VAS is a reliable and valid scale for patients with PFP [30]. Function will be assessed using the Patellofemoral Disorders Scale [31], which contains 13 questions that assess the severity of symptoms and limitations in different activities related to PFP. It presents a score between 0 to 100 points where the lower the score the worse the function. The Patellofemoral Disorders Scale is a reliable and valid scale for patients with PFP, which has been translated into Portuguese [30,31]. The kinematics of the lower limbs will be assessed through digital videos using two webcams (MyoVideo 139 HD Color Webcam) sampling at 30 frames per second and two leds (LED Floodlight) [32]. Reflective markers will be placed on the manubrium sterni and bilateral on the anterior superior iliac spine (ASIS), greater trochanter, lateral femoral epicondyle, fibular head and lateral malleolus [33]. All participants will be instructed to run at 10–12 km/hour on a motorized treadmill (Movement XL 1600). A treadmill acclimatization period of 6 min will be used before running kinematics are measured [34]. The frontal plane camera will be placed on a portable tripod perpendicular to the frontal plane at a height of 1.30m and a distance of 2.55m from the treadmill. The sagittal plane camera will be placed on a portable tripod, perpendicular to the sagittal plane at a height of 1.15m and a distance of 2.50m from the treadmill. The video recordings will be analyzed using the software MyoResearch 3.14—MyoVideo (Noraxon U.S.A. Inc.). In the frontal plane the angles assessed will be: contralateral pelvic drop; hip adduction [33,35,36]. These angles will be evaluated during midstance. In the sagittal plane the angles assessed will be: foot inclination; tibia inclination; ankle dorsiflexion; knee flexion [33,37,38]. Foot and tibia inclination will be evaluated during initial contact while ankle dorsiflexion and knee flexion during midstance. In the frontal plane, the deepest landing position (near midstance) will be determined visually by slowly advancing the video frame by frame [33,37,38]. Since the cameras are synchronized this position will also be used to determine the midstance in the sagittal plane. Initial contact will be determined visually by slowly advancing the video frame by frame, and will be defined as the first time that the foot touched the ground [37]. To analyze the proposed angles, seven steps will be considered [33]. The study of Dingenen and colleagues [39] has found excellent inter-rater reliability (ICC) for all angles with ICC’s ranging from 0.93 to 0.99.

### Rehabilitation programs (independent variables)

Each runner will take part in one of the three groups, two partially supervised gait retraining groups, one with a focus on impact (Group A), the other with a focus on cadence (Group B), and a control group (Group C). Before starting the intervention, participants will run with their regular shoes at a speed of 10/12 km/hour [40], in order to verify the usual values of the acceleration of tibia and cadence. After this assessment, it is possible to establish the threshold of impact and cadence that will be used in the programs. Both parameters will be acquired with the accelerometer Tgforce (v2.0.0.10) [20,41]. Also, these parameters will be collected immediately and six-months after the protocol to see how the participants have adapted to the proposed running-pattern. In groups A and B, the protocol will be performed four times a week, with a gradual duration from 15 to 30 minutes, over two weeks. There will be two supervised (first and fifth session) and six unsupervised sessions. In the final 4 training sessions the feedback will be gradually removed (Table 1). We chose a faded feedback design since it prevents the dependency on external feedback, promotes the development of response accuracy and consistency (important components of skill learning) and produce long-term retention [42]. In addition, participants will be instructed to run with this new running-pattern during the next six months.

**Table 1:** Protocol performed in groups A and B.

Week	Day	Training	Time of feedback	Time of training
1	1	Supervised	15 minutes	15 minutes
	2	Unsupervised	18 minutes	18 minutes
	3	Unsupervised	21 minutes	21 minutes
	4	Unsupervised	24 minutes	24 minutes
2	5	Supervised	21 minutes	27 minutes
	6	Unsupervised	15 minutes	30 minutes
	7	Unsupervised	9 minutes	30 minutes
	8	Unsupervised	3 minutes	30 minutes

Source: Authors.

Group A participants will receive visual feedback (acceleration of the tibia captured using an accelerometer) and verbal feedback (commands given by the clinician) throughout the intervention period. The accelerometer Tgforce (v2.0.0.10) will be taped to the anteromedial aspect of the subject’s distal tibia [20]. The study of Pairot de Fontenay and colleagues [41] assessed the concurrent validity of popular, commercially available wearable sensors in estimating vertical ground reaction force during running, and found that the Tgforce was the only wearable sensor to be valid in estimating instantaneous vertical loading rate. A screen positioned in front of the treadmill will show a graph of the acceleration of the tibia in real time captured by the accelerometer. On the screen, the participant will see a line that represents approximately 50% of the average peak tibial acceleration obtained during the last minute of running [19,20]. Subjects will be instructed to “run softer,” “make their footfalls quieter”, and to keep the acceleration peaks below the line [20,21,43]. Group B participants, on the other hand, will receive guidance regarding their cadence and will run with the help of a metronome with an adjusted cadence increased by 7.5 to 10% [44–46]. In both groups, the clinician will provide instructions repeatedly during the first minute, and provide positive or negative reinforcement approximately every 30 seconds for the rest of feedback period [43]. Participants will be instructed to maintain the new running pattern during the other unsupervised sessions held at a location of their choice [22]. Group C participants will not receive any retraining strategies or guidance until the study ends. After the 24-week follow-up, participants will receive guidance relevant to their condition based on biomechanical assessments previously performed.

Regarding the participants’ adherence, to ensure that they carry out the protocols and that they are executed properly, the supervised session will be carried out first. Other measures adopted by the researchers will be: (I) guidance after the supervised session on how the unsupervised sessions should take place; (II) give a copy of Table 1 containing information’s regarding the program and showing how unsupervised sessions should take place; (III): daily text message on the participants’ phone advising them on how the sessions should take place; (IV) inform the participants that they can contact the researchers to answer questions about the protocol.

Participants will be allowed to continue performing running activities during the study. There will be no need for the presence of a data monitoring committee or interim analyses and audits in order to determine the continuation or end of the study as the time for executing the protocol/study is short and the risks offered by it are minimal. The monitoring of possible adverse events will be done daily during the protocol period, any participant who reports pain worsening or other musculoskeletal pain will be instructed to reduce their training volume. The adverse effects will be addressed descriptively in the study results. If the pain persists to the point where participation is impossible, the participant will be considered as a drop-out of the intervention because of injury and the previous data collected will be used in the analysis.

### Outcomes measures (dependent variables)

The primary outcome of the study will be: (I) pain during daily activities (usual)—assessed using the Visual Analog Pain Scale [29]; The secondary outcomes will be: (II) pain during running—assessed using the Visual Analog Pain Scale [29]; (III) function—assessed using the Patellofemoral Disorders Scale [31]; (IV) kinematics of the lower limbs in the frontal (contralateral pelvic drop; hip adduction) [33,35,36] and sagittal planes (foot inclination; tibia inclination; ankle dorsiflexion; knee flexion) [33,37,38]–assessed through digital videos using two webcams (MyoVideo 139 HD Color Webcam) sampling at 30 frames per second and two leds (LED Floodlight) [33]. The video recordings will be analyzed using the MyoResearch 3.14 –MyoVideo software (Noraxon U.S.A. Inc.). The kinematic variables of interest were chosen based on previous studies [14,15,19,47,48]. The systematic review and meta-analysis of Neal and colleagues [14] shows a relationship between increased hip adduction and contralateral pelvic drop with PFP. The study of Wille and colleagues [47] showed that foot inclination angle and peak of knee flexion are variables that may predict knee joint loading and PFJ forces during running. Tibia inclination during initial contact is related with cadence and length [48] and these are associated with PFJ forces [18,19]. For the comparison of the three study subgroups, the average of points on the visual analog scale of pain, average of points on the scale of Patellofemoral disorders and the peak in degrees of the contralateral pelvic drop, hip adduction, knee flexion and ankle dorsiflexion during midstance and foot inclination and tibia inclination during the initial contact will be considered. The three groups will be compared before (t0), immediately after (t2) and six months (t24) after starting the protocol.

**Fig 1:**
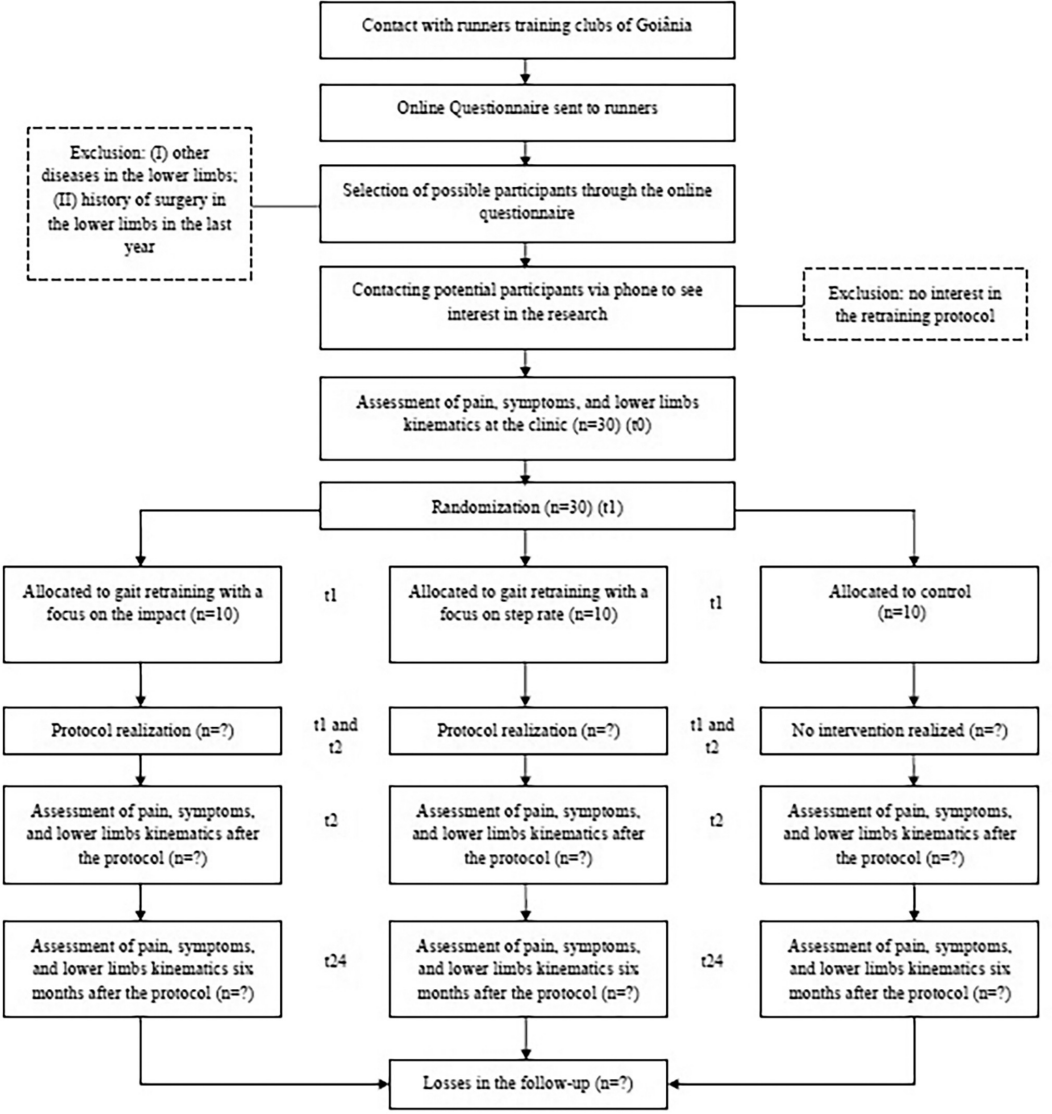
Diagram of the study.

Regarding the retention of the participants, to ensure that there are minimal losses during the follow-up period, the researchers will make a monthly phone call to monitor pain, the current level of training, physical activity practice and if the participants sought for other methods of treatment. Participants can withdraw from the study for any reason at any time, in which case the researchers will collect the pain and function outcomes using the aforementioned instruments (if possible) and report the reasons why these patients did not complete the follow-up (Fig 1).

### Data analysis

The data of each participant will be extracted for SPSS (Statistical Package for Social Sciences) version 23.0 for later statistical analysis. Descriptive analysis will be performed with calculation of means, standard deviations and 95% confidence intervals for quantitative variables and frequencies and percentages for qualitative variables. Data normality and sphericity will be tested using the Shapiro-Wilk and Mauchly’s tests, respectively. Baseline demographic data will be compared using ANOVA (parametric data), Kruskall-Wallis (non-parametric data) and Chi-squared tests. To compare the effects of the rehabilitation programs on the outcomes of the study, a repeated measures ANOVA with a 3-by-3 mixed-model (parametric data) or the Generalized Estimation Equations procedure with gamma distribution model (non-parametric data) will be used. Time (t0 x t2 x t24) will be used as repeated factor, group (impact x cadence x control) as independent factor and the mean of points on the visual analog pain scale, mean of points on the Patellofemoral Disorders Scale, and the peak in degrees of the contralateral pelvic drop, hip adduction, ankle dorsiflexion and knee flexion during midstance, and foot inclination and tibia inclination during initial contact as dependent variables. Baseline characteristics that are different across groups will be included as covariates in the model. Significance level of p <0.05 will be adopted. Sidak’s post-hoc will be used to make pairwise comparisons. Subgroup analysis and adjusted analysis will not be performed. Intention-to-treat analyses will be conducted where the subjects will be analyzed independently of adherence to the protocol or retention in the follow-up.

## Discussion

Running is increasingly popular since it demands minimal resources, however, it is an activity that has high injury rates [4,5]. A recent study showed that 2 million people adhered to running in 4 years in the Netherlands, on the other hand, the number of running-related injuries almost doubled in the same period [49]. Injuries generate immediate health costs with medical attendance or rehabilitation and future costs due to diseases related to physical inactivity, such costs impact directly runners and the health care system. In this context, PFP is one of the main running injuries, duration of pain and worst functionality is related with bad prognosis and unfortunately, more than half of patients present unfavorable recovery in long-terms [7,8]. In clinical practice, it is important to use interventions with known efficacy in reducing PFP, in order to maximize outcomes [8].

Despite recent systematic reviews about gait retraining, few adequately powered high-quality randomized clinical trials have been performed. Also the classic gait retraining protocols consume time and financial resources from the patient and therapist, since they need to be available to perform 8 supervised sessions within two/three weeks and minimally the clinic will need instruments to assess and display the real time kinematic or kinetic curves in a video monitor placed in front of a treadmill [22,50]. Another point is the applicability of the protocols since they are made in a controlled environment and not with the conditions that the participants usually run.

The use of other strategies such as a mirror in front of the treadmill [51], metronomes [44,45] and accelerometers [41] are recommended to diminish costs, however, do not resolve the time and applicability problem if do not made in an partially supervised format and outside the lab. The protocol that will be performed by group A requires a wearable device that can be given to the participant and pre-adjusted by the therapist. Participants can see the graph of the acceleration of the tibia in real time on the screen of the cellphone or can use headphones and receive an auditory input generated by the accelerometer while running [41]. The protocol that will be performed by group B requires a metronome that can be easily found for free in cellphone stores and can be pre-adjusted by the therapist. Participants can use headphones and hear the auditory input generated by the metronome while running [44,45]. We believe that performing the classic protocol in an unsupervised format, with the strategies listed above and outside the lab, will promote retention of the results generated for these kind of programs and help improve care in runners with PFP, while maximizing clinical applicability as well as time and cost-effectiveness.

## Supporting information

S1 File SPIRIT—administrative information. (DOCX)

S2 File Informed consent form (original version–Portuguese). (DOCX)

S3 File Informed consent form (translated version–English). (DOC)

S4 File SPIRIT—ethical aspects and dissemination of the results. (DOC)

S5 File SPIRIT—checklist. (DOC)

S6 File Study protocol. (PDF)

S1 Data (SAV)
