# Supplementary material for: Effects of gait retraining with focus on impact versus gait retraining with focus on cadence on pain, function and lower limb kinematics in runners with patellofemoral pain: Protocol of a randomized, blinded, parallel group trial with 6-month follow-up
Source: PLoS One. 2021 May 12;16(5):e0250965. doi: 10.1371/journal.pone.0250965 (PMC8116042; doi:10.1371/journal.pone.0250965)
Supplement: S1 File — (DOCX) [file pone.0250965.s001.docx]

**Title**

Effects of gait retraining with focus on impact versus gait retraining with focus on cadence on pain, function and lower limb kinematics in runners with patellofemoral pain: protocol of a randomized, blinded, parallel group trial with 6-month follow-up.

**Trial Registration**

Brazilian Registry of Clinical Trials / *Registro Brasileiro de Ensaios Clínicos* (REBEC)

RBR-8yb47v

**Protocol version**

Version 1.

19/11/2019.

Authors: JRSJ; PHRR.

**Funding**

This study is financed in part by the Coordenação de Aperfeiçoamento de Pessoal de Nível Superior – Brasil (CAPES) – Finance Code 001. Organizational costs may be requested to University of Brasília, Brasília – Brazil. Instituto Trata, Goiânia - Brazil will supply its physical structure and the necessary equipment to carry out the gait retraining protocol and the evaluations before, after and six months after the training. The remaining costs of the study will be financed by the primary researchers. The study design, collection, analysis and documentation of the study results will be entirely carried out by the main researchers.

**Roles and responsibilities - contributorship**

JRSJ (University of Brasilia, Brazil); PHRR (University of Brasilia, Brazil); TVL (State University of Goias, Brazil); JFE (The Running Clinic, and the University of British Columbia, Canada); JPSC (University of Brasilia, Brazil); JPCM (University of Brasilia, Brazil).

JRSJ: Conceptualization; Funding acquisition; Methodology; Project Administration; Resources; Writing – original draft; Writing – review & editing; PHRR: Conceptualization; Methodology; Project Administration; Resources; Writing – original draft; Writing – review & editing; TVL: Conceptualization; Methodology; Resources; Supervision; Writing – review & editing; JFE: Methodology; Supervision; Writing – review & editing; JPSC: Methodology; Writing – review & editing; JPCM: Conceptualization; Methodology; Resources; Supervision; Writing – review & editing.

**Roles and responsibilities - sponsor**

Trial Sponsor: University of Brasilia / *Universidade de Brasília* (UnB).

Address: AE, QNN 14, Ceilândia Sul, Brasília, Distrito Federal – Brazil. 72220-401.

Telephone: (+55) 61 3107-8400

**Roles and responsibilities – sponsor and funder** The financial support provided by the Coordenação de Aperfeiçoamento de Pessoal de Nível Superior – Brasil (CAPES) is a scholarship provided to the main author. The support from the University of Brasília and Instituto Trata - Goiânia are linked only to organizational costs, physical structure and equipment, and are not related to the study design, collection, analysis, writing and dissemination of the study results. The researchers in charge are solely responsible for the development of the study.

**Roles and responsibilities – committees**

*Researchers 1, 2 and 3 (JRSJ, PHRR and JPSC)*

Preparation of the protocol

Recruitment of participants

Randomization

Protocol execution (PHRR)

Data collection (JPSC)

Tracking follow-up (PHRR)

Organization of meetings with the steering committee

*Steering Committee (TVL, JFE and JPCM)*

Final opinion on the protocol

Review of study progress and, if necessary, changes to the protocol

Principal investigator advisor

*Data monitoring committee - not required*
