# Supplementary material for: Effects of gait retraining with focus on impact versus gait retraining with focus on cadence on pain, function and lower limb kinematics in runners with patellofemoral pain: Protocol of a randomized, blinded, parallel group trial with 6-month follow-up
Source: PLoS One. 2021 May 12;16(5):e0250965. doi: 10.1371/journal.pone.0250965 (PMC8116042; doi:10.1371/journal.pone.0250965)
Supplement: S2 File — (DOCX) [file pone.0250965.s002.docx]

**Ethical procedures**

This study was approved by the Research Ethics Committee (CEP) of the University of Brasilia / Faculdade de Ceilândia with decision number 4.132.491 and registered in the Brazilian Registry of Clinical Trials (REBEC) with number RBR-8yb47v. Any change in the protocol that may modify the execution of the study, potential benefits and risks for the participants, objectives, sample size, and study procedures will be formally addressed via addendum and only implemented after approval by the aforementioned Research Ethics Committee.

The Informed Consent Form (ICF) will be obtained by two researchers responsible for the study after explaining it to potential participants. In person, the researchers will explain the objectives, procedures, duration, risks, benefits, confidentiality of identity, and inform the voluntary nature of participation in the study. After the participant has communicated their understanding and agreement to the terms, they will sign the informed consent form.

The participants' data will be kept in individual and electronic folders. To guarantee the confidentiality of the information collected, both physical folders and electronic folders will be encrypted numerically to guarantee confidentiality of identity. The physical folders will be kept in a safe place, while the electronic folders will be accessed by the researchers on the laboratory computer using a password. To transmit the information among other researchers, the internet network or the laboratory's own external hardware will be used.

The authors of this manuscript have the following competing interests: JFE receives salary from The Running Clinic for teaching postgraduate continuing education to healthcare practitioners and providing lectures about running injury prevention to the public. This does not alter our adherence to PLoS One policies on sharing data and materials.

At the end of the follow-up period, if the participant's health is found to be impaired due to the protocol, the participant is guaranteed to carry out an evaluation and elaborate an intervention plan with two of the Physiotherapists linked to the study. Participants will in no way be compensated financially and such guarantee will be forfeited if it is found that such losses were due to negligence on the part of the same.

**Dissemination of the results**

As for the dissemination of the results, the participants will receive a complete individual report referring to the evaluations before, immediately, and six-months after the protocol, this will be sent via e-mail after the end of the follow-up period. For professionals and the community in general, the results will be disseminated through presentations at congresses and in scientific journals like PLoS One. The way of grouping the primary and secondary outcomes will be discussed together with the steering committee and each manuscript or abstract will only be submitted if approved. The authors will share the minimal data set necessary to replicate all study findings reported in the article. The deadline for submitting manuscripts from the study is estimated to be around 18 months after the randomization of the last participant.

Regarding the authorship of the study: JRSJ - Conceptualization; Funding acquisition; Methodology; Project Administration; Resources; Writing – original draft; Writing – review & editing; PHRR - Conceptualization; Methodology; Project Administration; Resources; Writing – original draft; Writing – review & editing; TVL - Conceptualization; Methodology; Resources; Supervision; Writing – review & editing; JFE - Methodology; Supervision; Writing – review & editing; JPSC - Methodology; Writing – review & editing; JPCM - Conceptualization; Methodology; Resources; Supervision; Writing – review & editing.
