# Supplementary material for: Effects of gait retraining with focus on impact versus gait retraining with focus on cadence on pain, function and lower limb kinematics in runners with patellofemoral pain: Protocol of a randomized, blinded, parallel group trial with 6-month follow-up
Source: PLoS One. 2021 May 12;16(5):e0250965. doi: 10.1371/journal.pone.0250965 (PMC8116042; doi:10.1371/journal.pone.0250965)
Supplement: S3 File — (DOC) [file pone.0250965.s003.doc]

***Termo de Consentimento Livre e Esclarecido - TCLE***

Convidamos o (a) Senhor (a) a participar do projeto de pesquisa **“Efeitos de dois programas de retreinamento de corrida sobre a dor, função e cinemática dos membros inferiores de corredores com dor patelofemoral: ensaio clínico randomizado com follow-up de seis meses”**, sob a responsabilidade do pesquisador **José Roberto de Souza Júnior**. O projeto será constituído pela aplicação de escalas específicas, por uma avaliação biomecânica e por programas de retreinamento de corrida. O retreinamento de corrida será feito por duas semanas, sendo que as escalas e a avaliação biomecânica serão realizadas antes, imediatamente e seis meses após o programa.

O objetivo desta pesquisa é determinar a influência de exercícios específicos de corrida na esteira sobre a dor, função e padrão de corrida de corredores com dor anterior no joelho. Se você tem dor, você está apto para participar desta pesquisa caso corra com o retropé, tenha cadência menor que 170 passos por minuto, idade entre 18 e 45 anos, apresente dor anterior no joelho acima de 3 pontos em uma escala de dor durante a corrida e em pelo menos uma atividade entre agachar, subir e descer degraus, ajoelhar e estender o joelho com resistência, e treine/corra com velocidade média entre 10-12 Km/hora.

A sua participação se dará por meio da coleta dos seus dados após a assinatura deste Termo de Consentimento Livre e Esclarecido. Inicialmente você responderá uma ficha de identificação para ver se está apto a entrar no estudo, caso esteja, os pesquisadores entrarão em contato e será agendada a sua avaliação. Após a avaliação você será em seguida sorteado para um dos grupos da pesquisa. Após o sorteio, os pesquisadores irão lhe informar as respectivas datas do tratamento, caso você tenha sido sorteado para tal, e as datas das reavaliações. Você deverá comparecer no local supracitado nos dias agendados com roupa apropriada para a prática de corrida. No dia da avaliação, iremos posicionar marcadores reflexíveis na região anterior do tronco, coxa, joelho, perna e tornozelo e você será filmado enquanto corre em uma esteira, na velocidade que você treina diariamente, para avaliação dos ângulos executados pelo seu tronco e pernas durante a corrida, após essa etapa serão realizados testes físico-funcionais para avaliar diferentes aspectos como mobilidade, flexibilidade e força muscular. A sua dor e função do joelho serão avaliados por meio de questionários específicos. Nos dias do tratamento, você será convidado a correr em uma esteira na velocidade que você treina diariamente enquanto segue instruções dos pesquisadores do estudo. De forma associada, você recebera instruções sobre como realizar esse mesmo tratamento em local de sua preferência. As reavaliações irão ocorrer exatamente da mesma forma que foram feitas as avaliações iniciais. As avaliações bem como os exercícios na esteira serão realizadas no Instituto Trata situado na Rua T-53, 1043 – Setor Bueno, Goiânia, Goiás, CEP: 74215-150. O tempo estimado para entendimento das informações contidas nesse TCLE bem como da aplicação da ficha de identificação será de 10 a 15 minutos. A avaliação na esteira dura em torno de 10 minutos enquanto que a avaliação física dura em torno de 15 minutos. O tratamento (para os sorteados para estes grupos) terá duração de duas semanas, sendo quatro vezes por semana durante no máximo 30 minutos. As reavaliações terão a mesma duração da avaliação inicial, 10 minutos para a avaliação na esteira e 15 para a avaliação física.

Os riscos decorrentes de sua participação na pesquisa envolvem a presença de desconforto cardiorrespiratório, neste caso o teste ou treino será interrompido e você será encaminhado para a unidade de saúde básica, e aumento da dor na região anterior do joelho ou em outras regiões das pernas, neste caso o teste ou treino será interrompido e os pesquisadores em conjunto irão realizar uma avaliação fisioterapêutica para identificar se tais dores ocorreram pelo protocolo e se você é elegível para continuar no estudo. Para minimizar tais riscos os pesquisadores irão avaliar estes sintomas durante a sessão e diariamente durante o protocolo. Se você aceitar participar, estará contribuindo para a utilização do treino de feedback, na funcionalidade de corredores com dor anterior no joelho. A alta incidência de lesões em corredores traz prejuízos na prática esportiva como redução da participação e do desempenho no esporte além de prejuízos financeiros. O sucesso do protocolo permitirá que os atletas tenham uma melhor funcionalidade e dessa forma menores chances de apresentarem lesões.

O (a) Senhor (a) pode se recusar a responder (ou participar de qualquer procedimento) qualquer questão que lhe traga constrangimento, podendo desistir de participar da pesquisa em qualquer momento sem nenhum prejuízo para o (a) senhor (a). O (a) Senhor receberá todos os esclarecimentos necessários antes e no decorrer da pesquisa e lhe será assegurado que seu nome não aparecerá sendo mantido o mais rigoroso sigilo pela omissão total de quaisquer informações que permitam identificá-lo. De forma similar, as imagens obtidas para avaliação da cinemática dos membros inferiores serão utilizadas somente para fins acadêmicos sendo garantida a ocultação da sua identidade, incluindo a região da face e/ou dos olhos, quando possível. Os pesquisadores não irão utilizar as imagens para fins comerciais ou com objetivos diversos da pesquisa proposta, sob pena de responsabilização nos termos da legislação brasileira.

Não há despesas pessoais para o participante em qualquer fase do estudo, incluindo valores das avaliações e dos programas de exercícios realizados. Também não há compensação financeira relacionada à sua participação, que será voluntária. Se existir qualquer despesa adicional relacionada diretamente à pesquisa (tais como, passagem para o local da pesquisa, alimentação no local da pesquisa ou exames para realização da pesquisa) a mesma será absorvida pelo orçamento da pesquisa.

Caso haja algum dano direto ou indireto decorrente de sua participação na pesquisa, você deverá buscar ser indenizado, obedecendo-se as disposições legais vigentes no Brasil.

Os resultados da pesquisa serão divulgados na Universidade de Brasília (UnB) podendo ser publicados posteriormente. Os dados e materiais serão utilizados somente para esta pesquisa e ficarão sob a guarda do pesquisador por um período de cinco anos, após isso serão destruídos.

Se o (a) Senhor (a) tiver qualquer dúvida em relação à pesquisa, por favor, telefone para: José Roberto de Souza Júnior ou Pedro Henrique Reis Rabelo nos telefones (62)982099360 / (62) 982056471 / (62)982371900, disponível inclusive para ligação a cobrar. O contato também poderá ser feito via e-mail: [joserobertofisio@gmail.com](mailto:joserobertofisio@gmail.com) ou [pedroreisrabelo@gmail.com](mailto:pedroreisrabelo@gmail.com).

Este projeto foi aprovado pelo Comitê de Ética em Pesquisa da Faculdade de Ceilândia (CEP/FCE) da Universidade de Brasília. O CEP é composto por profissionais de diferentes áreas cuja função é defender os interesses dos participantes da pesquisa em sua integridade e dignidade e contribuir no desenvolvimento da pesquisa dentro de padrões éticos. As dúvidas com relação à assinatura do TCLE ou os direitos do participante da pesquisa podem ser esclarecidos pelo telefone (61) 3107-8434 ou do e-mail cep.fce@gmail.com, horário de atendimento das 14h:00 às 18h:00, de segunda a sexta-feira. O CEP/FCE se localiza na Faculdade de Ceilândia, Sala AT07/66 – Prédio da Unidade de Ensino e Docência (UED) – Universidade de Brasília - Centro Metropolitano, conjunto A, lote 01, Brasília - DF. CEP: 72220-900.

Caso concorde em participar, pedimos que assine este documento que foi elaborado em duas vias, uma ficará com o pesquisador responsável e a outra com o Senhor (a).

______________________________________________

Nome / assinatura

____________________________________________

Pesquisador Responsável

José Roberto de Souza Júnior

Matrícula: 19/0007001

____________, ___ de __________de _________.
