# Supplementary material for: Effects of gait retraining with focus on impact versus gait retraining with focus on cadence on pain, function and lower limb kinematics in runners with patellofemoral pain: Protocol of a randomized, blinded, parallel group trial with 6-month follow-up
Source: PLoS One. 2021 May 12;16(5):e0250965. doi: 10.1371/journal.pone.0250965 (PMC8116042; doi:10.1371/journal.pone.0250965)
Supplement: S4 File — (DOC) [file pone.0250965.s004.doc]

***Informed Consent Form (ICF)***

We are inviting you to participate in the research project entitled **“Effects of two gait retraining programs on pain, functionality and lower limb kinematics in runners with patellofemoral pain: a randomized clinical trial with 6-months follow-up”**, under responsibility of the researcher José Roberto de Souza Júnior. The research protocol will be constituted of specific scales, biomechanics assessment, and gait retraining programs. The gait retraining will be done over two weeks, while the specific scales and the biomechanical assessment will be apply before, immediately after and six-months after the program.

The main purpose of this research will be to determine the influence of specific running exercises on pain, function and running pattern of runners with anterior knee pain. If you have anterior knee pain, you are able to participate in this study if you have age between 18 and 45 years old, run with your rearfoot with cadence under 170 steps/minute, being comfortable running at a speed of 10/12 km/hour, and present minimum pain levels of 3/10 on the Visual Analogue Scale (VAS) during running and one task among squatting, climbing, and descending steps, kneeling, and extending the knee with resistance.

You are able to participate in this study after the assignment of this Informed Conset Form. After this you will answer two specific scales, perform the biomechanical assessment and sorted into one of the three groups. To perform the biomechanical assessments the researchers will put reflexive markers in the anterior part of your trunk, thigh, knee, shin and ankle and you be recorded while running on a treadmill at a speed of 10/12 km/hour. These markers will be put in order to measure specific angles during running. Mobility and muscle strength tests will also be performed. If you have been sorted into one of the two gait retraining programs, the main researches will get in touch to schedule two dates for performing the program. On these dates you will run on a treadmill following specific commands given by the researches and you will receive instructions about how to perform the same protocol in a local of you preference over two weeks. If you have not been sorted for one of the two gait retraining programs, the researches will schedule the date for re-assessments. The re-assessments will occur in the same manner as the initial assessments.

The evaluations and the running exercises will be performed at the Instituto Trata located in the T-53 street, 1043, Sector Bueno, Goiânia, Goiás, ZIP: 74215-150. The estimated time to understand the information contained in this ICF will be 10 to 15 minutes. The symptoms and biomechanical assessment lasts around 10 minutes while the physical evaluation lasts around 15 minutes. The intervention (for those selected for these groups) will last for two weeks, four times a week for a maximum of 30 minutes. The re-assessment will have the same duration as the initial evaluation, 10 minutes for the evaluation on the biomechanical assessment and 15 for the physical evaluation.

The risks arising from your participation in the research involve the presence of cardiorespiratory discomfort, in which case the test or training will be interrupted and you will be referred to the basic health unit, and increased pain in the anterior region of the knee or in other regions of the lower limbs, in this case, the test or training will be interrupted and the researchers will carry out a physical therapy assessment to identify whether such pain occurred through the protocol and whether you are eligible to continue the study. To minimize such risks, researchers will assess these symptoms during the session and daily during the protocol. If you accept to participate, you will be contributing to the use of gait retraining, in the functionality of runners with previous knee pain. The high incidence of injuries in runners brings losses in the practice of sports such as reduced participation and performance in sport in addition to financial losses. The success of the protocol will allow athletes to have better functionality and keep practicing the sport.

You can refuse to answer (or participate in any procedure) any question that makes you uncomfortable, and you can give up participating in the research at any time without any harm to you. You will receive all necessary clarifications before and during the research and you will be assured that your name will not appear and the strictest confidentiality will be kept due to the total omission of any information that allows you to be identified. Similarly, the images obtained to evaluate the kinematics of the lower limbs will be used only for academic purposes and the concealment of their identity, including the region of the face and/or eyes, when possible, is guaranteed. Researchers will not use the images for commercial purposes or for purposes other than the proposed research, under penalty of liability under Brazilian law.

There are no personal expenses for the participant at any stage of the study, including values of assessments and exercise programs performed. There is also no financial compensation related to your participation, which will be voluntary. If there is any additional expense directly related to the research (such as travel to the research site, food at the research site or exams for conducting the research) it will be absorbed by the research budget.

If there is any direct or indirect damage resulting from your participation in the research, you must seek compensation, in compliance with the legal provisions in force in Brazil.

The research results will be released at the University of Brasília (UnB) and can be published later. The data and materials will be used only for this research and will be kept by the researcher for a period of five years, after which they will be destroyed.

If you have any questions regarding the research, please call: José Roberto de Souza Júnior or Pedro Henrique Reis Rabelo on the phones +5562982099360 / +5562982056471 / +5562982371900, available even for connection to demand. Contact can also be made via email: joserobertofisio@gmail.com or pedroreisrabelo@gmail.com.

This project was approved by the Research Ethics Committee of the Faculty of Ceilândia (RCE/FCE) of the University of Brasília. REC consists of professionals from different areas whose function is to defend the interests of research participants in their integrity and dignity and to contribute to the development of research within ethical standards.

Questions regarding the signature of the informed consent form or the rights of the research participant can be clarified by phone +55613107-8434 or by email cep.fce@gmail.com, office hours from 2:00 pm to 6:00 pm , from Monday to Friday. The RCE/FCE is located at the Faculty of Ceilândia - University of Brasília - Metropolitan Center, set A, lot 01, Brasília – DF, Brazil. ZIP: 72220-900.

If you agree to participate, we ask that you sign this document, which was prepared in two copies, one will be with the responsible researcher and the other with you.

______________________________________________

Name/signature

____________________________________________

Responsible Researcher

José Roberto de Souza Júnior

Registration: 19/0007001

____________, ___ de __________de _________.
