# Supplementary material for: Effects of gait retraining with focus on impact versus gait retraining with focus on cadence on pain, function and lower limb kinematics in runners with patellofemoral pain: Protocol of a randomized, blinded, parallel group trial with 6-month follow-up
Source: PLoS One. 2021 May 12;16(5):e0250965. doi: 10.1371/journal.pone.0250965 (PMC8116042; doi:10.1371/journal.pone.0250965)
Supplement: S6 File — (PDF) [file pone.0250965.s006.pdf]

NEWS | ABOUT | HELP | CONTACT

Search trials

ADVANCED SEARCH

HOME / REGISTERED TRIALS /

RBR-8yb47v
Effects of two gait retraining programs on pain, functionality and lower limb kinematics in runners with patellofemoral pain: a randomized clinical trial with 6-months follow-up.

Registration Date: Nov. 12, 2019, 12:44 p.m.

Last Update: July 6, 2020, 11:12 a.m.

Study Type:

Intervention Study

Scientific Title:

pt-BR	EN
Efeitos de dois programas de retreinamento de corrida sobre a dor, função e cinemática dos membros inferiores de corredores com dor patelofemoral: ensaio clínico randomizado com follow-up de seis meses.	Effects of two gait retraining programs on pain, functionality and lower limb kinematics in runners with patellofemoral pain: a randomized clinical trial with 6-months follow-up.

Trial Identification

UTN Number: U1111-1243-4811

Public Title:

pt-BR	EN
Efeitos de dois programas de exercícios sobre a dor, função e padrão de corrida de corredores com dor anterior no joelho	Effects of two exercise programs on pain, functionality and running pattern in runners with anterior knee pain

Scientific Acronym:

Public Acronym:

Secondary Identifying Numbers:

22631019.7.0000.8093
Issuing Authority: Plataforma Brasil

4.132.491
Issuing Authority: UnB - Faculdade de Ceilândia da Universidade de Brasília

Sponsors

Primary Sponsor: Universidade de Brasília

Secondary Sponsors:

Institution: Universidade de Brasília

Source(s) of Monetary or Material Support:

1

Online

Institution: Universidade de Brasília

Health Conditions

Health Condition(s) or Problem(s):

pt-BR
Condromalácia da rótula

EN
Chondromalacia patellae

General Descriptors for Health Condition(s):

pt-BR
M00-M99: XIII - Doenças do sistema osteomuscular e do tecido conjuntivo

EN
M00-M99: XIII - Diseases of the musculoskeletal system and connective tissue

Specific Descriptors for Health Condition(s):

pt-BR
M22.4: Condromalácia da rótula

ES
M22.4: Condromalacia de la rótula

EN
M22.4: Chondromalacia patellae

Interventions

Intervention Code(s)

Other

Interventions:

pt-BR
Grupo A (n=10) (Retreinamento de corrida com foco no impacto): o protocolo de retreinamento de corrida será realizado quatro vezes por semana, com duração gradativa de 15 para 30 minutos, durante duas semanas. Serão duas sessões de forma supervisionada e as outras seis de forma não supervisionada. Durante as sessões, os participantes irão correr calçados (tênis que utilizam habitualmente) em uma velocidade de 10-12 km/hora (velocidade de treino/confortável) e receberão um feedback visual (aceleração da tíbia captada por meio de um acelerômetro - Tgforce v2.0.0.10) e verbal (comandos dados pelos pesquisadores) durante todo o período da intervenção. Uma televisão posicionada em frente da esteira irá mostrar um gráfico da aceleração da tíbia em tempo real captado pelo acelerômetro. Na tela o participante irá ver uma linha que representa aproximadamente 50% da média do pico de aceleração da tíbia obtido durante o último minuto de corrida. O examinador irá orientar o participante a correr mais suave, tornar os passos mais silenciosos e manter o pico de aceleração da tíbia abaixo da

EN
Group A (n=10) (Gait retraining with focus on impact): the gait retraining protocol will be made 4 times a week, with a gradual duration of 15 to 30 minutes, for two weeks. It will be two supervised sessions and six non-supervised sessions. During the sessions, participants will run with their habitual shoes at a speed of 10-12 Km/hour (training speed/comfortable) and will receive visual feedback (acceleration of the tibia captured using an accelerometer - Tgforce v2.0.0.10) and verbal feedback (commands given by the clinician) throughout the intervention period. A screen positioned in front of the treadmill will show a graph of the acceleration of the tibia in real-time captured by the accelerometer. On the screen, the participant will see a line that represents approximately 50% of the average peak tibial acceleration obtained during the last minute of running. Subjects will be instructed to “run softer,” “make their footfalls quieter”, and to keep the acceleration peaks below the line. Participants will be instructed to maintain the new running pattern during the other unsupervised sessions held at a location of their choice.

linha mostrada na tela. Os participantes serão orientados a manter o novo padrão de corrida durante as demais sessões não supervisionadas realizadas em local de sua preferência

Grupo B (n=10) (Retreinamento de corrida com foco na cadência): o protocolo de retreinamento de corrida será realizado quatro vezes por semana, com duração gradativa de 15 para 30 minutos, durante duas semanas. Serão duas sessões de forma supervisionada e as outras seis de forma não supervisionada. Durante as sessões, os participantes irão correr calçados (tênis que utilizam habitualmente) em uma velocidade de 10-12 km/hora (velocidade de treino/confortável), receberão orientações com relação a sua cadência e irão correr com o auxílio de um metrônomo com uma cadência ajustada entre 7.5 a 10%. Os participantes serão orientados a manter o novo padrão de corrida durante as demais sessões não supervisionadas realizadas em local de sua preferência.

Grupo C (n=10) (Controle): este grupo não receberá quaisquer estratégias de retreinamento de corrida ou orientações até o término do estudo. Após essa etapa os participantes receberão orientações pertinentes a sua condição baseadas nas avaliações biomecânicas previamente realizadas.

Group B (n=10) (Gait Retraining with cadence focus): the gait retraining protocol will be made 4 times a week, with gradual duration of 15 to 30 minutes, during two weeks. It will be two supervisioned sessions and six non-supervisioned sessions. During the sessions, participants will run with their habitual shoes at a speed of 10-12 Km/hour (training speed/comfortable), will receive orientations in relation to their cadence and will run with a adjusted cadence of 7.5-10% with the guidance of a metronome. Participants will be instructed to maintain the new running pattern during the other unsupervised sessions held at a location of their choice.

Group C (n=10)(control): this group will not receive any gait retraining strategies or orientations until the end of the study. After the second evaluation participants will receive pertinent orientations related to their condition based on the previously biomechanical evaluations performed.

Descriptor for Intervention(s):

eT-BR

G11.427.410.568.610: Corrida

ES

G11.427.410.568.610: Carrera

Recruitment

Recruitment Status: Not yet recruiting

Recruitment Country
Brazil

Planned Date of First Enrollment: 2020-08-15

Planned Date of Last Enrollment: 2020-11-15

Target Sample Size:	Gender (inclusion sex):	Inclusion Minimum Age:	Inclusion Maximum Age:
30	-	18 Y	45 Y

Inclusion Criteria:

eT-BR

Corredores de rua de retropé com cadência menor que 170 passos por minuto, com

EU

Rearfoot runners with step rate under 170 steps/minute, age between 18 and 45

idade entre 18 e 45 anos, que apresentem dor anterior no joelho acima de 3 pontos na Escala Visual Analógica (EVA) durante a corrida e em pelo menos uma atividade entre agachar, subir e descer degraus, ajoelhar e estender o joelho de forma resistida, e que treinem/corram com velocidade média entre 10-12 Km/hora.

years, minimum pain levels of 3/10 on the Visual Analogue Scale (VAS) during running and one task among squatting, climbing, and descending steps, kneeling, and extending the knee with resistance, and being comfortable running at a speed of 10-12 km/hour.

Exclusion Criteria:

eT-BR

Outras doenças nos membros inferiores, antecedentes cirúrgicos no último ano e que não tenham interesse para realizar um programa de retreinamento de 2 semanas.

EU

Other diseases in the lower limbs, history of surgery in the lower limbs in the last year, and not showing interest to adhere to a strict running retraining protocol for 2 weeks.

Study Type

Study Design:

eT-BR

Ensaio clínico de tratamento, randomizado-controlado, paralelo, cego, com três braços

EU

Randomized-controlled, paralel, blind, three-arm treatment clinical trial

Expanded access program	Study Purpose	Intervention Assignment	Number of arms	Masking type	Allocation type	Study Phase
None	Treatment	Parallel	3	Single-blind	Randomized-controlled	N/A

Outcomes

Primary Outcomes:

eT-BR

Dor: será avaliada por meio da Escala Visual Analógica da Dor (EVA) que consiste de uma escala numérica de 0 a 10 pontos, onde 0 significa ausência de dor e 10 significa o máximo de dor já vivenciada. Será considerada a dor usual e dor durante a corrida. A dor será avaliada antes e após a intervenção.

EU

Pain: it will be evaluated through the Visual Analogue Scale (VAS) that consists of a numeric scale of 0 to 10 points, where 0 means absence of pain and 10 means the greatest pain experienced. It will be considered the usual pain and running pain. Pain will be evaluated before and after the intervention.

eT-BR

Função: será avaliada por meio do Questionário de Desordens Patelofemorais que consiste de um questionário traduzido e validado para a língua portuguesa que contém 13 questões que avaliam a severidade dos sintomas e a limitação em diferentes atividades relacionadas à dor patelofemoral. Apresenta uma pontuação entre 0 a 100 onde quanto menor a pontuação pior a função. A função será avaliada antes e após a intervenção.

EU

Functionality: it will be evaluated through the Scoring Patellofemoral Disorders Questionnaire that consists of a questionnaire translated and validated into the Portuguese language containing 13 questions that assess the severity of the symptoms and the limitation in different activities related to patellofemoral pain. Presents a score of 0 to 100, where lower scores refer to the worst functionality. The functionality will be evaluated before and after the intervention.

eT-BR

Cinemática dos membros inferiores durante a corrida: será avaliada por meio de vídeos digitais usando duas webcams (MyoVideo 139 HD Color Webcam) de amostragem a 30 quadros por segundo e dois leds (LED Floodlight). Marcadores reflexivos (19 mm) serão colocados no manúbrio do esterno e bilateralmente na Espinha Ilíaca Ântero-superior, trocânter maior, epicôndilo lateral do fêmur, cabeça da fíbula e maléolo lateral. Todos os participantes serão instruídos a correr em 10-12 km / hora em uma esteira motorizada (Movement XL 1600). A câmera do plano frontal será colocada em um tripé portátil perpendicular ao plano frontal, a uma altura de 1,30m e a uma distância de 2,55m da esteira. A câmera do plano sagital será colocada em um tripé portátil, perpendicular ao plano sagital, a uma altura de 1,15m e a uma distância de 2,50m da esteira. Os vídeos serão analisados usando o software MyoResearch 3.14 - MyoVideo (Noraxon U.S.A. Inc.) No plano frontal, os ângulos avaliados serão: queda pélvica contralateral; adução do quadril. Esses ângulos serão avaliados durante o apoio médio. No plano sagital, os ângulos avaliados serão: inclinação do pé; inclinação da tíbia; dorsiflexão do tornozelo; flexão do joelho (dois primeiros serão avaliados durante o contato inicial e os dois últimos no apoio médio). No plano frontal, a posição de aterrissagem mais profunda será determinada visualmente, avançando lentamente o vídeo frame por frame. Como as câmeras são sincronizadas essa posição também será usada para determinar o apoio médio no plano sagital. O contato inicial será determinado visualmente, avançando lentamente o vídeo, frame por frame, e será definido como a primeira vez que o pé tocou o chão. Para analisar os ângulos propostos, serão consideradas sete passos. A cinemática será avaliada antes e após a intervenção.

EU

Lower limbs Kinematics during running: will be assessed through digital videos using two webcams (MyoVideo 139 HD Color Webcam) sampling at 30 frames per second and two leds (LED Floodlight). Reflective markers will be placed on the manubrium sterni and bilateral on the anterior superior iliac spine (ASIS), greater trochanter, lateral femoral epicondyle, fibular head and lateral malleolus. All participants will be instructed to run in 10-12 km/ hour on a motorized treadmill (Movement XL 1600). The frontal plane camera will be placed on a portable tripod perpendicular to the frontal plane at a height of 1.30m and a distance of 2.55m from the treadmill. The sagittal plane camera will be placed on a portable tripod, perpendicular to the sagittal plane at a height of 1.15m, and a distance of 2.50m from the treadmill. The video recordings will be analyzed using the software MyoResearch 3.14 - MyoVideo (Noraxon U.S.A. Inc.) In the frontal plane the angles assessed will be: contralateral pelvic drop; hip adduction. These angles will be evaluated during the midstance. In the sagittal plane the angles assessed will be: foot inclination; tibia inclination; ankle dorsiflexion; knee flexion (the first two will be evaluated during initial contact while the last two during midstance). In the frontal plane, the deepest landing position (near midstance) will be determined visually by slowly advancing the video frame by frame. Since the cameras are synchronized this position will also be used to determine the midstance in the sagittal plane. Initial contact will be determined visually by slowly advancing the video frame by frame, and will be defined as the first time that the foot touched the ground. To analyze the proposed angles seven steps will be considered. Lower limb kinematics will be evaluated before and after the intervention.

Secondary Outcomes:

eT-BR

Dor: será avaliada por meio da Escala Visual Analógica da Dor (EVA) que consiste de uma escala numérica de 0 a 10 pontos, onde 0 significa ausência de dor e 10 significa o máximo de dor já vivenciada. Será considerada a dor usual, pior dor e dor durante a corrida. A dor será avaliada 6 meses após o protocolo.

EU

Pain: it will be evaluated through the Visual Analogue Scale (VAS) that consists of a numeric scale of 0 to 10 points, where 0 means absence of pain and 10 means the greatest pain experienced. It will be considered the usual pain, worst pain and running pain. Pain will be evaluated 6 months after the protocol.

pt-BR

Função: será avaliada por meio do Questionário de Desordens Patelofemorais que consiste de um questionário traduzido e validado para a língua portuguesa que contém 13 questões que avaliam a severidade dos sintomas e a limitação em diferentes atividades relacionadas à dor patelofemoral. Apresenta uma pontuação entre 0 a 100 onde quanto menor a pontuação pior a função. A função será avaliada 6 meses após o protocolo.

en

Functionality: it will be evaluated through the Scoring Patellofemoral Disorders Questionnaire that consists of a questionnaire translated and validated into the Portuguese language containing 13 questions that assess the severity of the symptoms and the limitation in different activities related to patellofemoral pain. Presents a score of 0 to 100, where lower scores refer to worst functionality. Functionality will be evaluated 6 months after the protocol.

pt-BR

Cinemática dos membros inferiores durante a corrida: será avaliada por meio de vídeos digitais usando duas webcams (MyoVideo 139 HD Color Webcam) de amostragem a 30 quadros por segundo e dois leds (LED Floodlight). Marcadores reflexivos (19 mm) serão colocados no manúbrio do esterno e bilateralmente na Espinha Ilíaca Ântero-superior, trocânter maior, epicôndilo lateral do fêmur, cabeça da fíbula e maléolo lateral. Todos os participantes serão instruídos a correr em 10-12 km / hora em uma esteira motorizada (Movement XL 1600). A câmera do plano frontal será colocada em um tripé portátil perpendicular ao plano frontal, a uma altura de 1,30m e a uma distância de 2,55m da esteira. A câmera do plano sagital será colocada em um tripé portátil, perpendicular ao plano sagital, a uma altura de 1,15m e a uma distância de 2,50m da esteira. Os vídeos serão analisados usando o software MyoResearch 3.14 - MyoVideo (Noraxon U.S.A. Inc.) No plano frontal, os ângulos avaliados serão: queda pélvica contralateral; adução do quadril. Esses ângulos serão avaliados durante o apoio médio. No plano sagital, os ângulos avaliados serão: inclinação do pé; inclinação da tíbia; dorsiflexão do tornozelo; flexão do joelho (dois primeiros serão avaliados durante o contato inicial e os dois últimos no apoio médio). No plano frontal, a posição de aterrissagem mais profunda será determinada visualmente, avançando lentamente o vídeo frame por frame. Como as câmeras são sincronizadas essa posição também será usada para determinar o apoio médio no plano sagital. O contato inicial será determinado

en

Lower limbs Kinematics during running: will be assessed through digital videos using two webcams (MyoVideo 139 HD Color Webcam) sampling at 30 frames per second and two leds (LED Floodlight). Reflective markers will be placed on the manubrium sterni and bilateral on the anterior superior iliac spine (ASIS), greater trochanter, lateral femoral epicondyle, fibular head and lateral malleolus. All participants will be instructed to run in 10-12 km/ hour on a motorized treadmill (Movement XL 1600). The frontal plane camera will be placed on a portable tripod perpendicular to the frontal plane at a height of 1.30m and a distance of 2.55m from the treadmill. The sagittal plane camera will be placed on a portable tripod, perpendicular to the sagittal plane at a height of 1.15m, and a distance of 2.50m from the treadmill. The video recordings will be analyzed using the software MyoResearch 3.14 - MyoVideo (Noraxon U.S.A. Inc.) In the frontal plane the angles assessed will be: contralateral pelvic drop; hip adduction. These angles will be evaluated during the midstance. In the sagittal plane the angles assessed will be: foot inclination; tibia inclination; ankle dorsiflexion; knee flexion (the first two will be evaluated during initial contact while the last two during midstance). In the frontal plane, the deepest landing position (near midstance) will be determined visually by slowly advancing the video frame by frame. Since the cameras are synchronized this position will also be used to determine the midstance in the sagittal plane. Initial contact will be determined visually by slowly advancing the video frame by frame, and

visualmente, avançando lentamente o vídeo, frame por frame, e será definido como a primeira vez que o pé tocou o chão. Para analisar os ângulos propostos, serão consideradas sete passos. A cinemática dos membros inferiores será avaliada 6 meses após o protocolo.

will be defined as the first time that the foot touched the ground. To analyze the proposed angles seven steps will be considered. Lower limb kinematics will be evaluated 6 months after the protocol.

Contacts

Contacts for Public Queries

Full Name: José Roberto de Souza Júnior

Address: Avenida Barro Preto Quadra 74 Lote 26 Jardim Imperial

City: Trindade / Brazil

Zip Code: 75380-000

Telephone: +5562982056471

E-mail: joserobertofisio@gmail.com

Affiliation: Universidade de Brasília

Contacts for Scientific Queries

Full Name: José Roberto de Souza Júnior

Address: Avenida Barro Preto Quadra 74 Lote 26 Jardim Imperial

City: Trindade / Brazil

Zip Code: 75380-000

Telephone: +5562982056471

E-mail: joserobertofisio@gmail.com

Affiliation: Universidade de Brasília

Full Name: Pedro Henrique Reis Rabelo

Address:

City: Brasília / Brazil

Zip Code: 72220-275

Telephone: +5562982371900

E-mail: pedroreisrabelo@gmail.com

Affiliation: Universidade de Brasília

Contact(s) for Site Queries

Full Name: José Roberto de Souza Júnior

Address: Avenida Barro Preto Quadra 74 Lote 26 Jardim Imperial

City: Trindade / Brazil

Zip Code: 75380-000

Telephone: +5562982056471

E-mail: joserobertofisio@gmail.com

Affiliation: Universidade de Brasília

Previous Revision

Additional Links:

Download as ICTRP format

Download as OpenTrials XML format

OpenTrials v1.2
